# Correction: Design and analysis of outcomes following SARS-CoV-2 infection in veterans

**DOI:** 10.1186/s12874-023-02021-4

**Published:** 2023-08-25

**Authors:** Valerie A. Smith, Theodore S. Z. Berkowitz, Paul Hebert, Edwin S. Wong, Meike Niederhausen, John A. Pura, Kristin Berry, Pamela Green, Anna Korpak, Alexandra Fox, Aaron Baraff, Alex Hickok, Troy A Shahoumian, Amy S.B. Bohnert, Denise M. Hynes, Edward J. Boyko, George N. Ioannou, Theodore J. Iwashyna, C. Barrett Bowling, Ann M. O’Hare, Matthew L. Maciejewski

**Affiliations:** 1 Center of Innovation to Accelerate Discovery and Practice Transformation, Durham VA Medical Center, Durham, NC USA; 2 Department of Population Health Sciences, Duke University, Durham, NC USA; 3 Division of General Internal Medicine, Department of Medicine, Duke University, Durham, NC USA; 4 Health Services Research & Development Center of Innovation for Veteran-Centered and Value- Driven Care, and Gastroenterology section, Veterans Affairs Puget Sound Health Care System, Seattle, WA USA; 5 Department of Health Systems and Population Health, University of Washington, Seattle, WA USA; 6 Center to Improve Veteran Involvement in Care, VA Portland Health Care System, Portland, OR USA; 7 Oregon Health & Science University (OHSU), Portland, OR USA; 8 Portland State University School of Public Health, Portland, OR USA; 9 Seattle Epidemiologic Research and Information Center, VA Puget Sound, Seattle, WA USA; 10 Population Health: Health Solutions, Veterans Health Administration, Washington, DC USA; 11 VA Center for Clinical Management Research, Ann Arbor, VA, MI USA; 12 Departments of Anesthesiology and Psychiatry, University of Michigan Medical School, Ann Arbor, MI USA; 13 College of Public Health and Human Sciences, Center for Quantitative Life Sciences, Oregon State University, Corvallis, OR USA; 14 Division of Gastroenterology, University of Washington, Seattle, WA USA; 15 National Clinical Scholars Program, University of Michigan Medical School, Ann Arbor, MI USA; 16 Department of Internal Medicine, University of Michigan Medical School, Ann Arbor, MI USA; 17 Geriatric Research Education and Clinical Center, Durham VA Medical Center, Durham, NC USA; 18 Department of Medicine, Duke University, Durham, NC USA; 19 Division of Nephrology, University of Washington, Seattle, WA USA

**Correction: BMC Medical Research Methodology 23, 81 (2023)**

10.1186/s12874-023-01882-z

Following the publication of the original article [1], the authors requested to update the number of covariates in Abstract section from *39* to *37* covariates. In section “Matching specification”, *39* has been changed to *37* (fourth paragraph), and “*count of CDC high-risk conditions, count of mental health conditions*,“ has been removed (fifth paragraph).

The original article [1] has been updated.
