# Supplementary material for: Design and analysis of outcomes following SARS-CoV-2 infection in veterans
Source: BMC Med Res Methodol. 2023 Apr 4;23:81. doi: 10.1186/s12874-023-01882-z (PMC10071454; doi:10.1186/s12874-023-01882-z)
Supplement: Supplementary file 1 — Supplementary Material 1 [file 12874_2023_1882_MOESM1_ESM.docx]

**Appendices for Design and Analysis of Outcomes following SARS-CoV-2 Infection in Veterans**

**Appendix Table 1. Original and Final List of Covariates for Matching**

| *Original Covariate List* | *Final Matching Specification Used* |
| --- | --- |
| Sex | Sex |
| Use of immunosuppressive medication | Use of immunosuppressive medication |
| Age | Age |
| Race | Race |
| Ethnicity | Ethnicity |
| CAN comorbidity score | CAN comorbidity score (7 categories) |
| Nosos comorbidity score | Nosos comorbidity score (11 categories) |
| Gagne comorbidity score indicators | Gagne comorbidity score |
| Indicator of CDC high-risk conditions (Cancer other than non-metastatic skin cancer) | Indicator of CDC high-risk conditions (Cancer other than non-metastatic skin cancer) |
| Indicator of CDC high-risk conditions (COPD/asthma/interstitial lung disease/cystic fibrosis/pulmonary) | Indicator of CDC high-risk conditions (COPD/asthma/interstitial lung disease/cystic fibrosis/pulmonary) |
| Indicator of CDC high-risk conditions (hypertension) | Indicator of CDC high-risk conditions (hypertension) |
| Indicator of CDC high-risk conditions (diabetes) | Indicator of CDC high-risk conditions (diabetes) |
| Indicator of CDC high-risk conditions (Dementia) | Indicator of CDC high-risk conditions (Dementia) |
| Indicator of CDC high-risk conditions (CAD) | Indicator of CDC high-risk conditions (CAD) |
| Indicator of CDC high-risk conditions (HIV) |  |
| Indicator of CDC high-risk conditions (Liver disease) | Indicator of CDC high-risk conditions (Liver disease) |
| Indicator of CDC high-risk conditions (Pregnancy) | Indicator of CDC high-risk conditions (Pregnancy)^1^ |
| Indicator of CDC high-risk conditions (Sickle cell/ thalassemia) | Indicator of CDC high-risk conditions (Sickle cell/ thalassemia) |
| Indicator of CDC high-risk conditions (Smoking) | Smoking status (4 categories) |
| Indicator of CDC high-risk conditions (Solid organ or blood stem cell transplant) | Indicator of CDC high-risk conditions (Solid organ or blood stem cell transplant) |
| Indicator of CDC high-risk conditions (Stroke or cerebrovascular disease) | Indicator of CDC high-risk conditions (Stroke or cerebrovascular disease) |
| Indicator of CDC high-risk conditions (CKD) | Indicator of CDC high-risk conditions (CKD) |
| Indicator of CDC high-risk conditions (CHF) | Indicator of CDC high-risk conditions (CHF) |
| Indicator of CDC high-risk conditions (SUD) | Indicator of CDC high-risk conditions (SUD) |
|  | Count of CDC high-risk conditions |
| SMI Diagnosis |  |
| Anxiety Diagnosis | Anxiety Diagnosis |
| PTSD Diagnosis | PTSD Diagnosis |
| Bipolar Diagnosis | Bipolar Diagnosis |
| Schizophrenia Diagnosis | Schizophrenia Diagnosis |
|  | Major depression Diagnosis |
|  | Count of mental health conditions |
| Body Mass Index (BMI) | Body Mass Index (BMI) |
| Community Living Center resident | Community Living Center resident |
| # VA telehealth visits |  |
| # VA in-person outpatient visits |  |
| # VA community care outpatient visits |  |
| # VA admissions | # admissions (VA + non-VA) |
|  | # VA primary care visits |
|  | # VA specialty care visits |
|  | # VA mental health care visits |
| State of residence | State of residence |
| Distance to nearest level 1a complexity VA facility | Distance to nearest VA medical center |
| Distance to nearest level 1b complexity VA facility |  |
| Distance to nearest level 1c complexity VA facility |  |
| Distance to nearest level 2 complexity VA facility |  |
| Distance to nearest level 3 complexity VA facility |  |
| Distance to nearest level CBOC |  |
| Rurality of residence | Rurality of residence (urban, rural) |
| Vaccinated for SARS-CoV-2 (January-April 2021) | Vaccinated for SARS-CoV-2 (January-April 2021) |
| Medicare enrolled (none, A only, A+B, A+B+D, MA) |  |

^1^Pregnancy indicator variable was constructed from ICD-10 diagnosis codes but was 0 for all patients in both arms of the study cohort.

Abbreviations: CAN: Care Assessment Need; CDC: Centers for Disease Control and Prevention; COPD: chronic obstructive pulmonary disease; CAD: coronary heart disease; HIV: human immunodeficiency virus; CKD: chronic kidney disease; CHF: congestive heart failure; SUD: substance use disorder; SMI: serious mental illness; PTSD: post-traumatic stress disorder; VA: Veterans Health Administration; CBOC: VA community-based outpatient clinic

**Appendix Table 2. Descriptive Statistics of Veterans with and without Matches**

| ***Variable Name*** | ***1+ Match***  (n=208,536) | ***Unable to Match***  (n=776) | ***SMD*** |
| --- | --- | --- | --- |
| **Age, mean (SD)** | 60.6 (16.2) | 54.0 (19.3) | .368 |
| **BMI, mean (SD)** | 31.3 (6.4) | 32.1 (7.2) | .117 |
| **Sex, N (%)** |  |  |  |
| Female | 21,949 (10.5) | 147 (18.9) | .704 |
| Male | 183,676 (88.1) | 482 (62.1) |  |
| Unknown | 2,911 (1.4) | 147 (18.9) |  |
| **Race, N (%)** |  |  |  |
| American Indian/Alaska Native | 1,965 (0.9) | 14 (1.8) | .514 |
| Asian | 2,081 (1.0) | 5 (0.6) |  |
| Black or African American | 47,726 (22.9) | 100 (12.9) |  |
| Native Hawaiian or Other Pacific Islander | 1,946 (0.9) | 13 (1.7) |  |
| White | 139,952 (67.1) | 472 (60.8) |  |
| Multiple Races | 1,978 (0.9) | 4 (0.5) |  |
| Missing | 12,888 (6.2) | 168 (21.6) |  |
| **Hispanic Ethnicity, N (%)** |  |  |  |
| Yes | 20,309 (9.7) | 77 (9.9) | .285 |
| No | 181,097 (86.8) | 617 (79.5) |  |
| Missing | 7,130 (3.4) | 82 (10.6) |  |
| **Rurality, N (%)** |  |  |  |
| Urban | 149,310 (71.6) | 466 (60.1) | .245 |
| Not Urban (incl. missing) | 59,226 (28.4) | 310 (39.9) |  |
| **Smoking Status, N (%)** |  |  |  |
| Current | 26,188 (12.6) | 72 (9.3) | .153 |
| Former | 88,275 (42.3) | 300 (38.7) |  |
| Never | 81,916 (39.3) | 353 (45.5) |  |
| Missing | 12,157 (5.8) | 51 (6.6) |  |
| **CDC High Risk Conditions, N (%)** |  |  |  |
| Coronary Heart Disease | 59,972 (28.8) | 180 (23.2) | .127 |
| Cancer | 38,136 (18.3) | 83 (10.7) | .217 |
| Chronic Kidney Disease | 47,610 (22.8) | 128 (16.5) | .160 |
| Congestive Heart Failure | 22,124 (10.6) | 70 (9.0) | .053 |
| Pulmonary | 46,178 (22.1) | 148 (19.1) | .076 |
| Dementia | 10,837 (5.2) | 49 (6.3) | .048 |
| Diabetes | 72,020 (34.5) | 201 (25.9) | .189 |
| Hypertension | 127,761 (61.3) | 360 (46.4) | .302 |
| Liver Disease | 15,324 (7.3) | 36 (4.6) | .114 |
| Sickle Cell | 383 (0.2) | 0 (0.0) | .061 |
| Transplant | 687 (0.3) | 5 (0.6) | .045 |
| Stroke/Cerebrovascular disease | 13,012 (6.2) | 40 (5.2) | .047 |
| Substance Use Disorder | 25,915 (12.4) | 49 (6.3) | .211 |
| Anxiety | 47,186 (22.6) | 195 (25.1) | .059 |
| Bipolar disorder | 8,039 (3.9) | 13 (1.7) | .133 |
| Major Depression | 67,383 (32.3) | 205 (26.4) | .130 |
| PTSD | 52,964 (25.4) | 138 (17.8) | .186 |
| Schizophrenia | 4,737 (2.3) | 15 (1.9) | .024 |
| **# CDC High Risk Conditions, mean (SD)** | 2.3 (1.9) | 1.7 (2.0) | .286 |
| **# Mental Health Conditions (Anxiety, Bipolar disorder, major depression, PTSD, schizophrenia), mean (SD)** | 0.9 (1.1) | 0.7 (1.0) | .131 |
| **Gagne score, mean (SD)** | 1.4 (2.3) | 1.1 (2.3) | .157 |
| **# VA Inpatient Admissions, mean (SD)** | 0.4 (1.2) | 0.2 (1.9) | .080 |
| **# VA Primary Care Visits, mean (SD)** | 8.6 (9.7) | 8.1 (13.5) | .040 |
| **# VA Specialty Care Visits, mean (SD)** | 13.9 (14.1) | 13.7 (27.0) | .010 |
| **Mental Health Care Utilization, mean (SD)** | 7.9 (22.6) | 4.7 (16.8) | .160 |
| **Immunosuppressed in prior 24 months, N (%)** | 20,366 (9.8) | 104 (13.4) | .114 |
| **CLC At Index Date, N (%)** | 2,176 (1.0) | 20 (2.6) | .115 |
| **NOSOS score, N (%)** |  |  | 5.102 |
| NOSOS, missing | 4,801 (2.3) | 735 (94.7) |  |
| NOSOS Category 1 [0, 0.417) | 5,708 (2.7) | 1 (0.1) |  |
| NOSOS Category 2 [0.417, 0.471) | 9,599 (4.6) | 0 (0.0) |  |
| NOSOS Category 3 [0.471, 0.534) | 12,449 (6.0) | 0 (0.0) |  |
| NOSOS Category 4 [0.534, 0.611) | 15,187 (7.3) | 0 (0.0) |  |
| NOSOS Category 5 [0.611, 0.707) | 17,737 (8.5) | 0 (0.0) |  |
| NOSOS Category 6 [0.707, 0.829) | 20,556 (9.9) | 0 (0.0) |  |
| NOSOS Category 7 [0.829, 0.998) | 23,462 (11.3) | 0 (0.0) |  |
| NOSOS Category 8 [0.998, 1.259) | 26,956 (12.9) | 1 (0.1) |  |
| NOSOS Category 9 [1.259, 1.805) | 31,372 (15.0) | 4 (0.5) |  |
| NOSOS Category 10 [1.805, Inf) | 40,709 (19.5) | 35 (4.5) |  |
| **CAN Score, N (%)** |  |  | 5.465 |
| CAN, missing | 4,066 (1.9) | 740 (95.4) |  |
| CAN Category 1, 0 – 20 | 34,427 (16.5) | 1 (0.1) |  |
| CAN Category 2, 25 – 40 | 31,926 (15.3) | 1 (0.1) |  |
| CAN Category 3, 45 – 60 | 38,266 (18.3) | 5 (0.6) |  |
| CAN Category 4, 65 – 80 | 46,894 (22.5) | 3 (0.4) |  |
| CAN Category 5, 85 – 90 | 30,745 (14.7) | 4 (0.5) |  |
| CAN Category 6, 95 – 99 | 22,212 (10.7) | 22 (2.8) |  |
| **Vaccinated in January-April 2021,** N (%) | 3,153 (1.5) | 495 (63.8) | .241 |
| **Index Month, N (%)** |  |  | .897 |
| March 2020 | 2,340 (1.1) | 0 (0.0) |  |
| April 2020 | 6,786 (3.3) | 7 (0.9) |  |
| May 2020 | 4,402 (2.1) | 2 (0.3) |  |
| June 2020 | 6,967 (3.3) | 1 (0.1) |  |
| July 2020 | 14,945 (7.2) | 2 (0.3) |  |
| August 2020 | 8,376 (4.0) | 7 (0.9) |  |
| September 2020 | 6,787 (3.3) | 9 (1.2) |  |
| October 2020 | 12,212 (5.9) | 184 (23.7) |  |
| November 2020 | 31,004 (14.9) | 182 (23.5) |  |
| December 2020 | 43,732 (21.0) | 101 (13.0) |  |
| January 2021 | 36,282 (17.4) | 92 (11.9) |  |
| February 2021 | 15,699 (7.5) | 75 (9.7) |  |
| March 2021 | 9,942 (4.8) | 60 (7.7) |  |
| April 2021 | 9,062 (4.3) | 54 (7.0) |  |
| **U.S. State of Residence, N (%)** |  |  |  |
| Alaska | 694 (0.3) | 6 (0.8) | 1.795 |
| Alabama | 4,089 (2.0) | 1 (0.1) |  |
| Arkansas | 3,075 (1.5) | 3 (0.4) |  |
| Arizona | 6,310 (3.0) | 7 (0.9) |  |
| California | 16,253 (7.8) | 1 (0.1) |  |
| Colorado | 3,278 (1.6) | 24 (3.1) |  |
| Connecticut | 1,895 (.9) | 10 (1.3) |  |
| Washington, D.C. | 414 (.2) | 2 (.3) |  |
| Delaware | 552 (.3) | 44 (5.7) |  |
| Florida | 16,003 (7.7) | 6 (.8) |  |
| Georgia | 7,893 (3.8) | 4 (.5) |  |
| Hawaii | 263 (.1) | 9 (1.2) |  |
| Iowa | 2,473 (1.2) | 91 (11.7) |  |
| Idaho | 1,582 (.8) | 7 (.9) |  |
| Illinois | 6,462 (3.1) | 27 (3.5) |  |
| Indiana | 4,533 (2.2) | 4 (.5) |  |
| Kansas | 2,471 (1.2) | 3 (.4) |  |
| Kentucky | 3,352 (1.6) | 4 (.5) |  |
| Louisiana | 3,754 (1.8) | 5 (.6) |  |
| Massachusetts | 2,990 (1.4) | 10 (1.3) |  |
| Maryland | 2,640 (1.3) | 16 (2.1) |  |
| Maine | 631 (.3) | 8 (1.0) |  |
| Michigan | 4,383 (2.1) | 8 (1.0) |  |
| Minnesota | 4,251 (2.0) | 16 (2.1) |  |
| Missouri | 6,609 (3.2) | 9 (1.2) |  |
| Mississippi | 1,804 (.9) | 1 (.1) |  |
| Montana | 1,122 (.5) | 3 (.4) |  |
| North Carolina | 8,521 (4.1) | 27 (3.5) |  |
| North Dakota | 865 (.4) | 10 (1.3) |  |
| Nebraska | 1,872 (.9) | 25 (3.2) |  |
| New Hampshire | 743 (.4) | 6 (.8) |  |
| New Jersey | 2,623 (1.3) | 11 (1.4) |  |
| New Mexico | 1,373 (.7) | 2 (.3) |  |
| Nevada | 3,049 (1.5) | 7 (.9) |  |
| New York | 7,739 (3.7) | 5 (.6) |  |
| Ohio | 9,173 (4.4) | 11 (1.4) |  |
| Oklahoma | 4,030 (1.9) | 7 (.9) |  |
| Oregon | 1,386 (.7) | 3 (.4) |  |
| Pennsylvania | 7,880 (3.8) | 16 (2.1) |  |
| Rhode Island | 762 (.4) | 4 (.5) |  |
| South Carolina | 6,352 (3.0) | 11 (1.4) |  |
| South Dakota | 1,408 (.7) | 253 (32.6) |  |
| Tennessee | 5,848 (2.8) | 253 (32.6) |  |
| Texas | 19,436 (9.3) | 4 (.5) |  |
| Utah | 1,243 (.6) | 1 (.1) |  |
| Virginia | 5,070 (2.4) | 2 (.3) |  |
| Vermont | 149 (.1) | 3 (.4) |  |
| Washington | 1,976 (.9) | 3 (.4) |  |
| Wisconsin | 5,087 (2.4) | 23 (3.0) |  |
| West Virginia | 1,621 (.8) | 2 (.3) |  |
| Wyoming | 554 (.3) | 5 (.6) |  |
| **Distance to Nearest VAMC (miles), mean (SD)** | 35.6 (36.5) | 37.1 (42.0) | .038 |

**Appendix Table 3. SARS-CoV-2 infected veterans and uninfected comparators prior to matching**

| ***Variable Name*** | ***COVID-19***  (n=209,312) | ***Uninfected***  (n=80,634,200) | ***SMD*** |
| --- | --- | --- | --- |
| **Age, mean (SD)** | 60.5 (16.2) | 62.1 (16.8) | .097 |
| **BMI, mean (SD)** | 31.3 (6.4) | 30.0 (6.0) | .223 |
| **Sex, N (%)** |  |  | .086 |
| Female | 22,096 (10.6) | 7,512,733 (9.3) |  |
| Male | 184,158 (88.0) | 71,068,138 (88.1) |  |
| Unknown | 3,058 (1.5) | 2,053,329 (2.5) |  |
| **Race, N (%)** |  |  | .130 |
| American Indian/Alaska Native | 1,979 (0.9) | 648,879 (0.8) |  |
| Asian | 2,086 (1.0) | 963,290 (1.2) |  |
| Black or African American | 47,826 (22.8) | 14,347,976 (17.8) |  |
| Native Hawaiian or Other Pacific Islander | 1,959 (0.9) | 675,091 (0.8) |  |
| White | 140,424 (67.1) | 57,917,844 (71.8) |  |
| Multiple Races | 1,982 (0.9) | 758,488 (0.9) |  |
| Missing | 13,056 (6.2) | 5,322,632 (6.6) |  |
| **Hispanic Ethnicity, N (%)** |  |  | .131 |
| Yes | 20,386 (9.7) | 5,058,390 (6.3) |  |
| No | 181,714 (86.8) | 72,234,780 (89.6) |  |
| Missing | 7,212 (3.4) | 3,341,030 (4.1) |  |
| **Rurality, N (%)** |  |  | .098 |
| Urban | 149,776 (71.6) | 54,063,501 (67.0) |  |
| Not Urban (incl. missing) | 59,536 (28.4) | 26,570,699 (33.0) |  |
| **Smoking Status, N (%)** |  |  | .258 |
| Current | 26,260 (12.5) | 14,238,094 (17.7) |  |
| Former | 88,575 (42.3) | 30,691,555 (38.1) |  |
| Never | 82,269 (39.3) | 26,727,400 (33.1) |  |
| Missing | 12,208 (5.8) | 8,977,151 (11.1) |  |
| **CDC High Risk Conditions, N (%)** |  |  |  |
| Coronary Heart Disease | 60,152 (28.7) | 18,241,909 (22.6) | .140 |
| Cancer | 38,219 (18.3) | 11,924,155 (14.8) | .094 |
| Chronic Kidney Disease | 47,738 (22.8) | 12,494,558 (15.5) | .187 |
| Congestive Heart Failure | 22,194 (10.6) | 5,087,069 (6.3) | .155 |
| Pulmonary | 46,326 (22.1) | 13,426,865 (16.7) | .139 |
| Dementia | 10,886 (5.2) | 2,093,909 (2.6) | .135 |
| Diabetes | 72,221 (34.5) | 20,821,842 (25.8) | .190 |
| Hypertension | 128,121 (61.2) | 43,830,338 (54.4) | .139 |
| Liver Disease | 15,360 (7.3) | 3,617,312 (4.5) | .121 |
| Sickle Cell | 383 (0.2) | 90,186 (0.1) | .019 |
| Transplant | 692 (.3) | 133,966 (0.2) | .033 |
| Stroke/Cerebrovascular disease | 13,052 (6.2) | 3,266,394 (4.1) | .099 |
| Substance Use Disorder | 25,964 (12.4) | 8,121,239 (10.1) | .074 |
| Anxiety | 47,381 (22.6) | 13,629,750 (16.9) | .144 |
| Bipolar disorder | 8,052 (3.8) | 2,339,917 (2.9) | .052 |
| Major Depression | 67,588 (32.3) | 19,216,275 (23.8) | .189 |
| PTSD | 53,102 (25.4) | 15,658,747 (19.4) | .143 |
| Schizophrenia | 4,752 (2.3) | 1,211,492 (1.5) | .056 |
| **# CDC High Risk Conditions, mean (SD)** | 2.3 (1.9) | 1.8 (1.7) | .288 |
| **# Mental Health Conditions (Anxiety, Bipolar disorder, major depression, PTSD, schizophrenia), mean (SD)** | 7.9 (22.6) | 5.0 (17.2) | .141 |
| **Gagne score, mean (SD)** | 1.4 (2.3) | .9 (1.8) | .267 |
| **# VA Inpatient Admissions, mean (SD)** | .4 (1.2) | .3 (1.3) | .087 |
| **# VA Primary Care Visits, mean (SD)** | 8.6 (9.7) | 5.5 (7.4) | .357 |
| **# VA Specialty Care Visits, mean (SD)** | 13.9 (14.2) | 8.7 (10.8) | .410 |
| **Mental Health Care Utilization, mean (SD)** | 7.9 (22.6) | 5.0 (17.2) | .141 |
| **Immunosuppressed in prior 24 months, N (%)** | 20,470 (9.8) | 4,139,682 (5.1) | .178 |
| **CLC At Index Date, N (%)** | 207,116 (99.0) | 80,323,908 (99.6) | .079 |
| **NOSOS score, N (%)** |  |  | .516 |
| NOSOS, missing | 5,536 (2.6) | 1,218,982 (1.5) |  |
| NOSOS Category 1 [0, 0.417) | 5,709 (2.7) | 7,950,900 (9.9) |  |
| NOSOS Category 2 [0.417, 0.471) | 9,599 (4.6) | 7,957,916 (9.9) |  |
| NOSOS Category 3 [0.471, 0.534) | 12,449 (5.9) | 7,949,509 (9.9) |  |
| NOSOS Category 4 [0.534, 0.611) | 15,187 (7.3) | 7,946,789 (9.9) |  |
| NOSOS Category 5 [0.611, 0.707) | 17,737 (8.5) | 7,944,176 (9.9) |  |
| NOSOS Category 6 [0.707, 0.829) | 20,556 (9.8) | 7,941,327 (9.8) |  |
| NOSOS Category 7 [0.829, 0.998) | 23,462 (11.2) | 7,938,423 (9.8) |  |
| NOSOS Category 8 [0.998, 1.259) | 26,957 (12.9) | 7,934,856 (9.8) |  |
| NOSOS Category 9 [1.259, 1.805) | 31,376 (15.0) | 7,930,401 (9.8) |  |
| NOSOS Category 10 [1.805, Inf) | 40,744 (19.5) | 7,920,921 (9.8) |  |
| **CAN Score, N (%)** |  |  | .419 |
| CAN, missing | 4,806 (2.3) | 330,773 (0.4) |  |
| CAN Category 1, 0 – 20 | 34,428 (16.4) | 21,417,817 (26.6) |  |
| CAN Category 2, 25 – 40 | 31,927 (15.3) | 16,603,294 (20.6) |  |
| CAN Category 3, 45 – 60 | 38,271 (18.3) | 15,772,164 (19.6) |  |
| CAN Category 4, 65 – 80 | 46,897 (22.4) | 15,293,253 (19.0) |  |
| CAN Category 5, 85 – 90 | 30,749 (14.7) | 7,505,220 (9.3) |  |
| CAN Category 6, 95 – 99 | 22,234 (10.6) | 3,711,679 (4.6) |  |
| **Vaccinated in January-April 2021,** N (%) | 138,046 (66.0) | 58,387,490 (72.4) | .245 |
| **Index Month, N (%)** |  |  | .813 |
| March 2020 | 2,340 (1.1) | 5,974,920 (7.4) |  |
| April 2020 | 6,793 (3.2) | 5,953,363 (7.4) |  |
| May 2020 | 4,404 (2.1) | 5,922,178 (7.3) |  |
| June 2020 | 6,968 (3.3) | 5,890,494 (7.3) |  |
| July 2020 | 14,947 (7.1) | 5,854,855 (7.3) |  |
| August 2020 | 8,383 (4.0) | 5,830,914 (7.2) |  |
| September 2020 | 6,796 (3.2) | 5,807,370 (7.2) |  |
| October 2020 | 12,396 (5.9) | 5,778,237 (7.2) |  |
| November 2020 | 31,186 (14.9) | 5,724,988 (7.1) |  |
| December 2020 | 43,833 (20.9) | 5,650,171 (7.0) |  |
| January 2021 | 36,374 (17.4) | 5,586,838 (6.9) |  |
| February 2021 | 15,774 (7.5) | 5,561,049 (6.9) |  |
| March 2021 | 10,002 (4.8) | 5,550,317 (6.9) |  |
| April 2021 | 9,116 (4.4) | 5,548,506 (6.9) |  |
| **U.S. State of Residence, N (%)** |  |  |  |
| Alaska | 700 (.3) | 254,961 (0.3) | .212 |
| Alabama | 4,090 (2.0) | 1,539,544 (1.9) |  |
| Arkansas | 3,078 (1.5) | 1,102,378 (1.4) |  |
| Arizona | 6,317 (3.0) | 2,159,188 (2.7) |  |
| California | 16,254 (7.8) | 6,203,098 (7.7) |  |
| Colorado | 3,302 (1.6) | 1,466,142 (1.8) |  |
| Connecticut | 1,905 (.9) | 640,717 (0.8) |  |
| Washington, D.C. | 416 (.2) | 101,353 (0.1) |  |
| Delaware | 596 (.3) | 234,740 (0.3) |  |
| Florida | 16,009 (7.6) | 7,195,109 (8.9) |  |
| Georgia | 7,897 (3.8) | 3,067,846 (3.8) |  |
| Hawaii | 272 (.1) | 390,312 (0.5) |  |
| Iowa | 2,564 (1.2) | 903,537 (1.1) |  |
| Idaho | 1,589 (.8) | 623,083 (0.8) |  |
| Illinois | 6,489 (3.1) | 2,242,322 (2.8) |  |
| Indiana | 4,537 (2.2) | 1,720,731 (2.1) |  |
| Kansas | 2,474 (1.2) | 783,795 (1.0) |  |
| Kentucky | 3,356 (1.6) | 1,334,547 (1.7) |  |
| Louisiana | 3,759 (1.8) | 1,174,934 (1.5) |  |
| Massachusetts | 3,000 (1.4) | 1,060,816 (1.3) |  |
| Maryland | 2,656 (1.3) | 1,180,726 (1.5) |  |
| Maine | 639 (0.3) | 547,677 (0.7) |  |
| Michigan | 4,391 (2.1) | 2,120,231 (2.6) |  |
| Minnesota | 4,267 (2.0) | 1,542,590 (1.9) |  |
| Missouri | 6,618 (3.2) | 1,872,865 (2.3) |  |
| Mississippi | 1,805 (0.9) | 876,222 (1.1) |  |
| Montana | 1,125 (0.5) | 497,508 (0.6) |  |
| North Carolina | 8,548 (4.1) | 3,321,561 (4.1) |  |
| North Dakota | 875 (0.4) | 265,625 (0.3) |  |
| Nebraska | 1,897 (0.9) | 610,243 (0.8) |  |
| New Hampshire | 749 (0.4) | 409,282 (0.5) |  |
| New Jersey | 2,634 (1.3) | 961,030 (1.2) |  |
| New Mexico | 1,375 (0.7) | 710,814 (0.9) |  |
| Nevada | 3,056 (1.5) | 1,056,982 (1.3) |  |
| New York | 7,744 (3.7) | 2,791,710 (3.5) |  |
| Ohio | 9,184 (4.4) | 3,155,471 (3.9) |  |
| Oklahoma | 4,037 (1.9) | 1,334,839 (1.7) |  |
| Oregon | 1,389 (.7) | 1,288,099 (1.6) |  |
| Pennsylvania | 7,896 (3.8) | 3,007,013 (3.7) |  |
| Rhode Island | 766 (.4) | 243,225 (0.3) |  |
| South Carolina | 6,363 (3.0) | 1,910,140 (2.4) |  |
| South Dakota | 1,661 (0.8) | 391,601 (0.5) |  |
| Tennessee | 5,854 (2.8) | 2,011,111 (2.5) |  |
| Texas | 19,440 (9.3) | 7,019,237 (8.7) |  |
| Utah | 1,244 (0.6) | 483,066 (0.6) |  |
| Virginia | 5,072 (2.4) | 2,309,550 (2.9) |  |
| Vermont | 152 (0.1) | 187,587 (0.2) |  |
| Washington | 1,979 (0.9) | 1,735,098 (2.2) |  |
| Wisconsin | 5,110 (2.4) | 1,610,339 (2.0) |  |
| West Virginia | 1,623 (0.8) | 731,742 (0.9) |  |
| Wyoming | 559 (0.3) | 251,863 (0.3) |  |
| **Distance to Nearest VAMC (miles), mean (SD)** | 35.6 (36.6) | 41.4 (38.3) | .154 |

Note: The uninfected cohort represents 6,245,364 unique Veterans with a total of 80,634,200 person-months because each individual contributed a person-month each month they were eligible.

**Appendix Table 4. List of immunosuppressant medications used to define baseline immunosuppressant drug status.**

| 'ABATACEPT 125MG/ML INJ,PEN,1ML', 'ABATACEPT 125MG/ML SYRINGE', 'ABATACEPT 250MG/VIL INJ', 'ADALIMUMAB 10MG/0.1ML INJ,SYRINGE,KIT', 'ADALIMUMAB 10MG/0.2ML INJ,SYRINGE,KIT', 'ADALIMUMAB 20MG/0.2ML INJ,SYRINGE,KIT', 'ADALIMUMAB 20MG/0.4ML INJ,SYRINGE,KIT', 'ADALIMUMAB 40MG/0.4ML INJ,PEN,KIT', 'ADALIMUMAB 40MG/0.4ML INJ,SYRINGE,KIT', 'ADALIMUMAB 40MG/0.4ML X 1/80MG/0.8ML X 1 INJ,SYRINGE,KIT', 'ADALIMUMAB 40MG/0.4ML X 2/80MG/0.8ML X 1 INJ,SYRINGE,KIT', 'ADALIMUMAB 40MG/0.8ML INJ,PEN,CROHNS STARTER PKG,6', 'ADALIMUMAB 40MG/0.8ML INJ,PEN,KIT', 'ADALIMUMAB 40MG/0.8ML INJ,PEN,PSORIASIS STARTER PKG,4', 'ADALIMUMAB 40MG/0.8ML INJ,SYRINGE,KIT', 'ADALIMUMAB 80MG/0.8ML INJ PEN KIT', 'ADALIMUMAB 80MG/0.8ML INJ,PEN,CD-UC-HS STARTER PKG,3', 'ADALIMUMAB 80MG/0.8ML INJ,SYRINGE, PED,CROHNS STARTER PKG,3', 'ALEMTUZUMAB 10MG/ML INJ,VIL,1.2ML', 'ALEMTUZUMAB 30MG/ML INJ', 'ANAKINRA 100MG/0.67ML INJ,SYRINGE', 'AZATHIOPRINE 100MG TAB', 'AZATHIOPRINE 100MG/VIL INJ', 'AZATHIOPRINE 50MG TAB', 'AZATHIOPRINE 75MG TAB', 'BASILIXIMAB 10MG/VIL INJ', 'BASILIXIMAB 20MG/VIL INJ', 'BELIMUMAB 120MG/VIL INJ', 'BELIMUMAB 200MG/ML INJ,PEN,1ML', 'BELIMUMAB 200MG/ML INJ,SYR,1ML', 'BELIMUMAB 400MG/VIL INJ', 'BENDAMUSTINE HCL 100MG/VIL INJ', 'BENDAMUSTINE HCL 25MG/ML INJ,VIL,4ML', 'BENDAMUSTINE HCL 25MG/VIL INJ', 'BETAMETHASONE ACETATE 3MG/BETAMETHASONE NA PO4 3MG/ML INJ,SUSP', 'BLEOMYCIN SO4 15UNT/VIL INJ', 'BLEOMYCIN SO4 30UNT/VIL INJ', 'BLINATUMOMAB 35MCG/VIL INJ', 'BOSUTINIB 100MG TAB', 'BOSUTINIB 400MG TAB', 'BOSUTINIB 500MG TAB', 'BUDESONIDE 3MG CAP,EC', 'BUDESONIDE 6MG CAP,SA', 'BUDESONIDE 9MG CAP,SA', 'BUDESONIDE 9MG TAB,SA', 'BUSULFAN 2MG TAB', 'BUSULFAN 6MG/ML INJ,AMP,10ML', 'BUSULFAN 6MG/ML INJ,VIL,10ML', 'CARBOPLATIN 10MG/ML INJ,SOLN', 'CARBOPLATIN 150MG/VIL INJ', 'CARMUSTINE 100MG/VIL INJ', 'CARMUSTINE 7.7MG IMPLANT WAFER', 'CERTOLIZUMAB PEGOL 200MG/ML INJ,SOLN,SYR,KIT,2', 'CERTOLIZUMAB PEGOL 200MG/ML INJ,SOLN,SYR,STARTER KIT,6', 'CERTOLIZUMAB PEGOL 200MG/VIL,INJ', 'CHLORAMBUCIL 2MG TAB', 'CISPLATIN 100MG/VIL (PF) INJ', 'CISPLATIN 1MG/ML INJ', 'CISPLATIN 200MG/VIL (PF) INJ', 'CISPLATIN 50MG/VIL (PF) INJ', 'CLADRIBINE 10MG TAB DOSEPAK,10', 'CLADRIBINE 10MG TAB DOSEPAK,5', 'CLADRIBINE 10MG TAB DOSEPAK,6', 'CLADRIBINE 10MG TAB DOSEPAK,7', 'CLADRIBINE 10MG TAB DOSEPAK,8', 'CLADRIBINE 10MG TAB DOSEPAK,9', 'CLADRIBINE 1MG/ML INJ', 'CLOZAPINE (CLOZARIL) 100MG TAB', 'CLOZAPINE (CLOZARIL) 100MG TAB,UD', 'CLOZAPINE (CLOZARIL) 25MG TAB', 'CLOZAPINE (CLOZARIL) 25MG TAB,UD', 'CLOZAPINE (MYLAN) 100MG TAB', 'CLOZAPINE (MYLAN) 100MG TAB,RAPID DISINTEGRATING', 'CLOZAPINE (MYLAN) 100MG TAB,UD', 'CLOZAPINE (MYLAN) 200MG TAB', 'CLOZAPINE (MYLAN) 200MG TAB,UD', 'CLOZAPINE (MYLAN) 25MG TAB', 'CLOZAPINE (MYLAN) 25MG TAB,RAPID DISINTEGRATING', 'CLOZAPINE (MYLAN) 25MG TAB,UD', 'CLOZAPINE (MYLAN) 50MG TAB', 'CLOZAPINE (TEVA) 100MG TAB', 'CLOZAPINE (TEVA) 100MG TAB,RAPID DISINTEGRATING', 'CLOZAPINE (TEVA) 100MG TAB,UD', 'CLOZAPINE (TEVA) 100MG TAB,UD,RAPID DISINTEGRATING', 'CLOZAPINE (TEVA) 12.5MG TAB,RAPID DISINTEGRATING', 'CLOZAPINE (TEVA) 150MG TAB,RAPID DISINTEGRATING', 'CLOZAPINE (TEVA) 150MG TAB,UD,RAPID DISINTEGRATING', 'CLOZAPINE (TEVA) 200MG TAB', 'CLOZAPINE (TEVA) 200MG TAB,RAPID DISINTEGRATING', 'CLOZAPINE (TEVA) 200MG TAB,UD,RAPID DISINTEGRATING', 'CLOZAPINE (TEVA) 25MG TAB', 'CLOZAPINE (TEVA) 25MG TAB,RAPID DISINTEGRATING', 'CLOZAPINE (TEVA) 25MG TAB,UD', 'CLOZAPINE (TEVA) 25MG TAB,UD,RAPID DISINTEGRATING', 'CLOZAPINE (TEVA) 50MG TAB', 'CLOZAPINE (VERSACLOZ) 50MG/ML SUSP,ORAL', 'CYCLOPHOSPHAMIDE 1GM/VIL INJ', 'CYCLOPHOSPHAMIDE 200MG/ML INJ,SOLN', 'CYCLOPHOSPHAMIDE 25MG CAP', 'CYCLOPHOSPHAMIDE 25MG TAB', 'CYCLOPHOSPHAMIDE 2GM/VIL INJ', 'CYCLOPHOSPHAMIDE 500MG/VIL INJ', 'CYCLOPHOSPHAMIDE 50MG CAP', 'CYCLOPHOSPHAMIDE 50MG TAB', 'CYCLOSPORINE (GENGRAF) 100MG CAP,UD', 'CYCLOSPORINE (GENGRAF) 100MG/ML SOLN,ORAL', 'CYCLOSPORINE (GENGRAF) 25MG CAP,UD', 'CYCLOSPORINE (NEORAL) 100MG CAP', 'CYCLOSPORINE (NEORAL) 25MG CAP', 'CYCLOSPORINE (SANDIMMUNE) 100MG CAP', 'CYCLOSPORINE (SANDIMMUNE) 100MG/ML SOLN,ORAL', 'CYCLOSPORINE (SANDIMMUNE) 25MG CAP', 'CYCLOSPORINE 0.09% (PF) SOLN,OPH,0.25ML', 'CYCLOSPORINE 50MG/ML INJ', 'CYCLOSPORINE MICROEMULSION 100MG/ML SOLN', 'CYCLOSPORINE MODIFIED 100MG CAP', 'CYCLOSPORINE MODIFIED 100MG/ML SOLN,ORAL', 'CYCLOSPORINE MODIFIED 25MG CAP', 'CYCLOSPORINE MODIFIED 50MG CAP', 'CYCLOSPORINE NON-MODIFIED 100MG CAP', 'CYCLOSPORINE NON-MODIFIED 100MG/ML SOLN,ORAL', 'CYCLOSPORINE NON-MODIFIED 25MG CAP', 'DASATINIB 100MG TAB', 'DASATINIB 140MG TAB', 'DASATINIB 20MG TAB', 'DASATINIB 50MG TAB', 'DASATINIB 70MG TAB', 'DASATINIB 80MG TAB', 'DEXAMETHASONE 0.2MG/ML/NACL 0.9% INJ,BAG,50ML', 'DEXAMETHASONE 0.5MG TAB', 'DEXAMETHASONE 0.5MG/5ML ELIXIR', 'DEXAMETHASONE 0.5MG/5ML SOLN,ORAL', 'DEXAMETHASONE 0.75MG TAB', 'DEXAMETHASONE 0.75MG TAB DOSEPAK,12', 'DEXAMETHASONE 1.5MG TAB', 'DEXAMETHASONE 1.5MG TAB DOSEPAK,21', 'DEXAMETHASONE 1.5MG TAB DOSEPAK,35', 'DEXAMETHASONE 1MG TAB', 'DEXAMETHASONE 1MG/1ML SOLN,ORAL', 'DEXAMETHASONE 20MG TAB', 'DEXAMETHASONE 2MG TAB', 'DEXAMETHASONE 2MG TAB,UD', 'DEXAMETHASONE 4MG TAB', 'DEXAMETHASONE 6MG TAB', 'DEXAMETHASONE 9% SUSP,INTRAOCULAR,KIT', 'DEXAMETHASONE ACETATE PWDR', 'DEXAMETHASONE NA PHOSPHATE (PF) 10MG/ML INJ,SOLN', 'DEXAMETHASONE NA PHOSPHATE 10MG/ML INJ,SOLN', 'DEXAMETHASONE NA PHOSPHATE 10MG/ML INJ,SOLN,SYRINGE,1ML', 'DEXAMETHASONE NA PHOSPHATE 4MG/ML INJ,SOLN', 'DEXAMETHASONE NA PHOSPHATE 4MG/ML INJ,SOLN,SYRINGE,1ML', 'DEXAMETHASONE SODIUM PHOSPHATE PWDR', 'DIMETHYL FUMARATE 120,14/240MG,46 CAP,EC', 'DIMETHYL FUMARATE 120MG CAP,EC', 'DIMETHYL FUMARATE 240MG CAP,EC', 'DOXORUBICIN HCL 10MG/VIL (PF) INJ', 'DOXORUBICIN HCL 2MG/ML INJ', 'DOXORUBICIN HCL 50MG/VIL (PF) INJ', 'DOXORUBICIN HCL LIPOSOME 2MG/ML INJ', 'ECULIZUMAB 10MG/ML INJ,SOLN', 'ETANERCEPT 25MG/0.5ML INJ SYRINGE', 'ETANERCEPT 25MG/VIL INJ W/SYRINGE', 'ETANERCEPT 50MG/ML INJ CARTRIDGE', 'ETANERCEPT 50MG/ML INJ SURECLICK', 'ETANERCEPT 50MG/ML INJ SYRINGE', 'ETOPOSIDE 20MG/ML INJ,SOLN', 'ETOPOSIDE 20MG/ML INJ,VIL,25ML', 'ETOPOSIDE 20MG/ML INJ,VIL,5ML', 'ETOPOSIDE 50MG CAP', 'ETOPOSIDE PHOSPHATE 100MG/VIL INJ', 'EVEROLIMUS 0.25MG TAB', 'EVEROLIMUS 0.5MG TAB', 'EVEROLIMUS 0.75MG TAB', 'EVEROLIMUS 10MG TAB', 'EVEROLIMUS 1MG TAB', 'EVEROLIMUS 2.5MG TAB', 'EVEROLIMUS 5MG TAB', 'EVEROLIMUS 5MG TAB,SUSP,ORAL,UD', 'EVEROLIMUS 7.5MG TAB', 'FINGOLIMOD 0.25MG CAP', 'FINGOLIMOD 0.5MG CAP', 'FLUDARABINE PO4 10MG TAB', 'FLUDARABINE PO4 25MG/ML INJ,VIL,2ML', 'FLUDARABINE PO4 50MG/VIL INJ', 'GLATIRAMER ACETATE 20MG/ML INJ,SYR,1ML,30', 'GLATIRAMER ACETATE 40MG/ML INJ,SYR,1ML', 'GOLIMUMAB 100MG/ML INJ SMARTJECT', 'GOLIMUMAB 100MG/ML INJ SYRINGE', 'GOLIMUMAB 12.5MG/ML INJ,VIL,4ML', 'GOLIMUMAB 50MG/0.5ML INJ SMARTJECT', 'GOLIMUMAB 50MG/0.5ML INJ SYRINGE', 'HYDROCORTISONE 100MG/60ML ENEMA', 'HYDROCORTISONE 10MG TAB', 'HYDROCORTISONE 20MG TAB', 'HYDROCORTISONE 5MG TAB', 'HYDROCORTISONE NA SUCCINATE 1000MG/VIL INJ,SOLN', 'HYDROCORTISONE NA SUCCINATE 100MG/VIL INJ,SOLN', 'HYDROCORTISONE NA SUCCINATE 250MG/VIL INJ,SOLN', 'HYDROCORTISONE NA SUCCINATE 500MG/VIL INJ,SOLN', 'HYDROCORTISONE NA SUCCINATE 50MG/ML INJ,SOLN', 'HYDROXYCHLOROQUINE SO4 200MG TAB', 'IBRUTINIB 140MG CAP,ORAL', 'IBRUTINIB 140MG TAB,ORAL', 'IBRUTINIB 280MG TAB,ORAL', 'IBRUTINIB 420MG TAB,ORAL', 'IBRUTINIB 560MG TAB,ORAL', 'IBRUTINIB 70MG CAP,ORAL', 'IDELALISIB 100MG TAB', 'IDELALISIB 150MG TAB', 'IFOSFAMIDE 1GM/VIL INJ', 'IFOSFAMIDE 3GM/VIL INJ', 'IFOSFAMIDE 50MG/ML INJ,20ML', 'IFOSFAMIDE 50MG/ML INJ,60ML', 'IMATINIB MESYLATE 100MG TAB', 'IMATINIB MESYLATE 400MG TAB', 'INFLIXIMAB 100MG/VIL (PF) INJ', 'INTERFERON BETA-1A 22MCG/0.5ML PEN INJ (REBIDOSE)', 'INTERFERON BETA-1A 22MCG/0.5ML SYR INJ (REBIF)', 'INTERFERON BETA-1A 44MCG/0.5ML PEN INJ (REBIDOSE)', 'INTERFERON BETA-1A 44MCG/0.5ML SYR INJ (REBIF)', 'INTERFERON BETA-1A 8.8MCG X 6/22MCG X 6 PEN INJ (REBIDOSE)', 'INTERFERON BETA-1A 8.8MCG X 6/22MCG X 6 SYR INJ (REBIF)', 'INTERFERON BETA-1A,RECOMBINANT 30MCG/0.5ML INJ,PEN (AVONEX)', 'INTERFERON BETA-1A,RECOMBINANT 30MCG/0.5ML INJ,SYRINGE (AVONEX)', 'INTERFERON BETA-1B,RECOMBINANT 0.3MG/VIL (BETASERON)', 'INTERFERON BETA-1B,RECOMBINANT 0.3MG/VIL (EXTAVIA)', 'LEFLUNOMIDE 10MG TAB', 'LEFLUNOMIDE 20MG TAB', 'LOMUSTINE 100MG CAP', 'LOMUSTINE 10MG CAP', 'LOMUSTINE 40MG CAP', 'MELPHALAN 2MG TAB', 'MELPHALAN FLUPHENAMIDE 20MG/VIL INJ', 'MELPHALAN HCL 50MG/VIL INJ', 'MERCAPTOPURINE 20MG/ML SUSP,ORAL', 'MERCAPTOPURINE 50MG TAB', 'METHOTREXATE 10MG/0.2ML AUTO INJECTOR', 'METHOTREXATE 10MG/0.4ML AUTO INJECTOR', 'METHOTREXATE 12.5MG/0.25ML AUTO INJECTOR', 'METHOTREXATE 12.5MG/0.4ML AUTO INJECTOR', 'METHOTREXATE 15MG/0.3ML AUTO INJECTOR', 'METHOTREXATE 15MG/0.4ML AUTO INJECTOR', 'METHOTREXATE 17.5MG/0.35ML AUTO INJECTOR', 'METHOTREXATE 17.5MG/0.4ML AUTO INJECTOR', 'METHOTREXATE 20MG/0.4ML AUTO INJECTOR', 'METHOTREXATE 22.5MG/0.45ML AUTO INJECTOR', 'METHOTREXATE 22.5MG/0.4ML AUTO INJECTOR', 'METHOTREXATE 25MG/0.4ML AUTO INJECTOR', 'METHOTREXATE 25MG/0.5ML AUTO INJECTOR', 'METHOTREXATE 30MG/0.6ML AUTO INJECTOR', 'METHOTREXATE 7.5MG/0.15ML AUTO INJECTOR', 'METHOTREXATE NA 10MG TAB', 'METHOTREXATE NA 1GM/VIL INJ', 'METHOTREXATE NA 2.5MG TAB', 'METHOTREXATE NA 2.5MG TAB,UD', 'METHOTREXATE NA 250MG/VIL INJ', 'METHOTREXATE NA 25MG/ML (PF) INJ', 'METHOTREXATE NA 25MG/ML INJ', 'METHOTREXATE NA 5MG TAB', 'METHOTREXATE SODIUM 2.5MG/ML SOLN,ORAL', 'METHYLPREDNISOLONE 16MG TAB', 'METHYLPREDNISOLONE 2MG TAB', 'METHYLPREDNISOLONE 32MG TAB', 'METHYLPREDNISOLONE 4MG TAB', 'METHYLPREDNISOLONE 4MG TAB DOSEPAK,21', 'METHYLPREDNISOLONE 4MG TAB,UD', 'METHYLPREDNISOLONE 8MG TAB', 'METHYLPREDNISOLONE ACETATE 20MG/ML INJ,SUSP', 'METHYLPREDNISOLONE ACETATE 40MG/ML INJ,SUSP', 'METHYLPREDNISOLONE ACETATE 80MG/ML INJ,SUSP', 'METHYLPREDNISOLONE NA SUCCINATE 1000MG/VIL INJ', 'METHYLPREDNISOLONE NA SUCCINATE 125MG/VIL INJ', 'METHYLPREDNISOLONE NA SUCCINATE 2000MG/VIL INJ', 'METHYLPREDNISOLONE NA SUCCINATE 40MG/VIL INJ', 'METHYLPREDNISOLONE NA SUCCINATE 500MG/VIL INJ', 'MUROMONAB-CD3 1MG/ML INJ,5ML', 'MYCOPHENOLATE MOFETIL (CELLCEPT) 1000MG/5ML SUSP,ORAL', 'MYCOPHENOLATE MOFETIL (CELLCEPT) 250MG CAP', 'MYCOPHENOLATE MOFETIL (CELLCEPT) 500MG TAB', 'MYCOPHENOLATE MOFETIL 1000MG/5ML SUSP,ORAL', 'MYCOPHENOLATE MOFETIL 250MG CAP', 'MYCOPHENOLATE MOFETIL 250MG CAP,UD', 'MYCOPHENOLATE MOFETIL 500MG TAB', 'MYCOPHENOLATE MOFETIL 500MG TAB,UD', 'MYCOPHENOLATE MOFETIL HYDROCHLORIDE (CELLCEPT) 500MG/VIL INJ,IV', 'MYCOPHENOLATE MOFETIL HYDROCHLORIDE 500MG/VIL INJ', 'MYCOPHENOLIC ACID 180MG TAB,EC', 'MYCOPHENOLIC ACID 360MG TAB,EC', 'NATALIZUMAB 20MG/ML INJ,SOLN,15ML', 'NILOTINIB HCL 150MG CAP,ORAL', 'NILOTINIB HCL 200MG CAP,ORAL', 'NILOTINIB HCL 50MG CAP,ORAL', 'OBINUTUZUMAB 25MG/ML INJ,VIL,40ML', 'OFATUMUMAB 20MG/0.4ML PEN INJ (SENSOREADY)', 'OFATUMUMAB 20MG/ML INJ', 'OLAPARIB 100MG TAB', 'OLAPARIB 150MG TAB', 'OMACETAXINE MEPESUCCINATE 3.5MG/VIL INJ,LYPHL', 'OXALIPLATIN 100MG/VIL (PF) INJ', 'OXALIPLATIN 50MG/VIL (PF) INJ', 'OXALIPLATIN 5MG/ML (PF) INJ,SOLN', 'PALBOCICLIB 100MG CAP,ORAL', 'PALBOCICLIB 100MG TAB', 'PALBOCICLIB 125MG CAP,ORAL', 'PALBOCICLIB 125MG TAB', 'PALBOCICLIB 75MG CAP,ORAL', 'PALBOCICLIB 75MG TAB', 'PENTOSTATIN 10MG/MANNITOL 50MG/VIL INJ', 'PONATINIB 10MG TAB', 'PONATINIB 15MG TAB', 'PONATINIB 30MG TAB', 'PONATINIB 45MG TAB', 'PREDNISOLONE 3MG/ML SOLN,ORAL', 'PREDNISOLONE 5MG TAB', 'PREDNISOLONE NA PHOSPHATE 10MG TAB,ORAL,DISINTEGRATING', 'PREDNISOLONE NA PHOSPHATE 10MG/5ML (A/F) SOLN,ORAL', 'PREDNISOLONE NA PHOSPHATE 15MG TAB,ORAL DISINTEGRATING', 'PREDNISOLONE NA PHOSPHATE 15MG/5ML SOLN,ORAL', 'PREDNISOLONE NA PHOSPHATE 20MG/5ML SOLN,ORAL', 'PREDNISOLONE NA PHOSPHATE 30MG TAB,ORAL DISINTEGRATING', 'PREDNISOLONE NA PHOSPHATE 5MG/5ML LIQUID,ORAL', 'PREDNISONE 10MG TAB', 'PREDNISONE 10MG TAB DOSEPACK,21', 'PREDNISONE 10MG TAB DOSEPACK,48', 'PREDNISONE 1MG TAB', 'PREDNISONE 1MG TAB,EC', 'PREDNISONE 2.5MG TAB', 'PREDNISONE 20MG TAB', 'PREDNISONE 20MG TAB,UD', 'PREDNISONE 2MG TAB,EC', 'PREDNISONE 50MG TAB', 'PREDNISONE 5MG TAB', 'PREDNISONE 5MG TAB DOSEPACK,48', 'PREDNISONE 5MG TAB DOSEPAK,21', 'PREDNISONE 5MG TAB,EC', 'PREDNISONE 5MG TAB,UD', 'PREDNISONE 5MG/5ML SOLN,ORAL', 'PREDNISONE 5MG/ML SOLN,CONC', 'PREDNISONE PWDR', 'RITUXIMAB 10MG/ML INJ,VIL,10ML', 'RITUXIMAB 10MG/ML INJ,VIL,50ML', 'SECUKINUMAB 150MG/ML INJ,PEN 1ML', 'SECUKINUMAB 150MG/ML INJ,SYR,1ML', 'SILTUXIMAB 100MG/VIL INJ', 'SILTUXIMAB 400MG/VIL INJ', 'SIROLIMUS 0.5MG TAB', 'SIROLIMUS 0.5MG TAB,UD', 'SIROLIMUS 1MG TAB', 'SIROLIMUS 1MG TAB,UD', 'SIROLIMUS 1MG/ML SOLN,ORAL', 'SIROLIMUS 2MG TAB', 'SULFASALAZINE 500MG TAB', 'SULFASALAZINE 500MG TAB,EC', 'TACROLIMUS (ASTAGRAF XL) 0.5MG CAP,SA', 'TACROLIMUS (ASTAGRAF XL) 1MG CAP,SA', 'TACROLIMUS (ASTAGRAF XL) 5MG CAP,SA', 'TACROLIMUS (ENVARSUS XR) 0.75MG TAB,SA', 'TACROLIMUS (ENVARSUS XR) 1MG TAB,SA', 'TACROLIMUS (ENVARSUS XR) 4MG TAB,SA', 'TACROLIMUS (PROGRAF) 0.2MG/PKT GRNL,RCNST-ORAL', 'TACROLIMUS (PROGRAF) 0.5MG CAP', 'TACROLIMUS (PROGRAF) 1MG CAP', 'TACROLIMUS (PROGRAF) 1MG/PKT GRNL,RCNST-ORAL', 'TACROLIMUS (PROGRAF) 5MG CAP', 'TACROLIMUS 0.5MG CAP', 'TACROLIMUS 1MG CAP', 'TACROLIMUS 5MG CAP', 'TACROLIMUS 5MG/ML INJ,SOLN,1ML AMP', 'THALIDOMIDE 100MG CAP', 'THALIDOMIDE 150MG CAP', 'THALIDOMIDE 200MG CAP', 'THALIDOMIDE 50MG CAP', 'THIOGUANINE 40MG TAB', 'TOCILIZUMAB 162MG/0.9ML AUTOINJECTOR', 'TOCILIZUMAB 162MG/0.9ML INJ,SYRINGE,0.9ML', 'TOCILIZUMAB 20MG/ML INJ,SOLN,10ML', 'TOCILIZUMAB 20MG/ML INJ,SOLN,20ML', 'TOCILIZUMAB 20MG/ML INJ,SOLN,4ML', 'TOFACITINIB 10MG TAB,ORAL', 'TOFACITINIB 11MG 24HR TAB,SA', 'TOFACITINIB 22MG 24HR TAB,SA', 'TOFACITINIB 5MG TAB,ORAL', 'TRIAMCINOLONE ACETONIDE 10MG/ML INJ,SUSP', 'TRIAMCINOLONE ACETONIDE 32MG/VIL INJ,SUSP,SA', 'TRIAMCINOLONE ACETONIDE 40MG/ML INJ,SUSP', 'TRIAMCINOLONE ACETONIDE 80MG/ML INJ,SUSP', 'TRIAMCINOLONE HEXACETONIDE 20MG/ML INJ,SUSP', 'USTEKINUMAB 45MG/0.5ML INJ,SYR,0.5ML', 'USTEKINUMAB 5MG/ML INJ,SOLN,26ML', 'USTEKINUMAB 90MG/ML INJ,SYR,1ML', 'VEDOLIZUMAB 300MG/VIL INJ', 'VINBLASTINE SO4 1MG/ML INJ', 'VINCRISTINE SO4 1MG/ML (PF) INJ', 'VINCRISTINE SO4 LIPOSOME 0.16MG/ML INJ,KIT', 'ZIV-AFLIBERCEPT 25MG/ML INJ,4ML', 'ZIV-AFLIBERCEPT 25MG/ML INJ,8ML' |
| --- |
